# Lacrimal Cytokines Assessment in Subjects Exposed to Different Levels of Ambient Air Pollution in a Large Metropolitan Area

**DOI:** 10.1371/journal.pone.0143131

**Published:** 2015-11-20

**Authors:** Monique Matsuda, Rodolfo Bonatti, Mônica V. Marquezini, Maria L. B. Garcia, Ubiratan P. Santos, Alfésio L. F. Braga, Milton R. Alves, Paulo H. N. Saldiva, Mário L. R. Monteiro

**Affiliations:** 1 Laboratory for investigation in Ophthalmology (LIM-33), University of Sao Paulo Medical School, Sao Paulo, SP, Brazil; 2 Laboratory of Experimental Air Pollution (LPAE), University of Sao Paulo Medical School, Sao Paulo, SP, Brazil; 3 Pulmonary Division—Heart Institute(InCor), Hospital das Clínicas da Faculdade de Medicina da Universidade de São Paulo, Sao Paulo, SP, Brazil; 4 Environmental Exposure and Risk Assessment Group, Collective Health Post-graduation Program, Catholic University of Santos, Santos, SP, Brazil; Boston University School of Medicine, UNITED STATES

## Abstract

**Background:**

Air pollution is one of the most environmental health concerns in the world and has serious impact on human health, particularly in the mucous membranes of the respiratory tract and eyes. However, ocular hazardous effects to air pollutants are scarcely found in the literature.

**Design:**

Panel study to evaluate the effect of different levels of ambient air pollution on lacrimal film cytokine levels of outdoor workers from a large metropolitan area.

**Methods:**

Thirty healthy male workers, among them nineteen professionals who work on streets (taxi drivers and traffic controllers, high pollutants exposure, Group 1) and eleven workers of a Forest Institute (Group 2, lower pollutants exposure compared to group 1) were evaluated twice, 15 days apart. Exposure to ambient PM_2.5_ (particulate matter equal or smaller than 2.5 μm) was 24 hour individually collected and the collection of tears was performed to measure interleukins (IL) 2, 4, 5 and 10 and interferon gamma (IFN-γ) levels. Data from both groups were compared using Student’s t test or Mann- Whitney test for cytokines. Individual PM_2.5_ levels were categorized in tertiles (lower, middle and upper) and compared using one-way ANOVA. Relationship between PM_2.5_ and cytokine levels was evaluated using generalized estimating equations (GEE).

**Results:**

PM_2.5_ levels in the three categories differed significantly (lower: ≤22 μg/m^3^; middle: 23–37.5 μg/m^3^; upper: >37.5 μg/m^3^; *p*<0.001). The subjects from the two groups were distributed unevenly in the lower category (Group 1 = 8%; Group 2 = 92%), the middle category (Group 1 = 89%; Group 2 = 11%) and the upper category (Group 1 = 100%). A significant relationship was found between IL-5 and IL-10 and PM_2.5_ levels of the group 1, with an average decrease of 1.65 pg/mL of IL-5 level and of 0.78 pg/mL of IL-10 level in tear samples for each increment of 50 μg/m3 of PM_2.5_ (*p* = 0.01 and *p* = 0.003, respectively).

**Conclusion:**

High levels of PM_2.5_ exposure is associated with decrease of IL-5 and IL-10 levels suggesting a possible modulatory action of ambient air pollution on ocular surface immune response.

## Introduction

Air pollution has been associated with a number of adverse health effects, mostly related to respiratory and cardiovascular alterations [1,2,3,4]. However, few studies have investigated the air pollution effects on the ocular surface despite the fact that the ocular mucosa is continuously exposed to the external environment [5,6,7,8,9,10,11,12,13].

Air pollution consists of a mixture of solid and liquid particles suspended in the air, including fine (PM_2.5_) and coarse (PM_2.5–10_) particulate matter, and different types of gases (ozone, nitrogen oxides, sulfur oxides, volatile organic carbons, hydrocarbons and carbon monoxide) mostly originating from motor vehicles and industries in developed urban centers [1].

Clinical studies have demonstrated increased rates of ocular symptoms, such as irritation, redness, teary eyes [6,7,9] and ocular surface and tear film abnormalities in healthy individuals exposed to acute [12,13] and chronic urban air pollution [11]. Furthermore, using impression cytology, Novaes et al. found goblet cell hyperplasia in the conjunctiva of subjects chronically exposed to high levels of traffic-related air pollution [10] and, more recently, Torricelli et al. encountered a negative correlation between PM_2.5_ and tear film osmolarity levels in a similar cohort [5]. Nevertheless, the immune mechanisms involved in the adverse effects of air pollution on the ocular surface remain largely unknown.

The lacrimal film contains thousands of components that play a pivotal role in antimicrobial defense, wound repair and inflammatory response in order to maintain normal homeostasis. Among such components, cytokines are essential molecules involved in the coordination of the inflammatory processes [14] and form an intricate signaling network crucial at different stages of the immune response promoted by T helper (Th) lymphocytes involved in the activation, proliferation, and death of pathogens. Th lymphocytes may be classified into subtypes (such as Th1, Th2 and Th17) according to their functional capacities and cytokine patterns. Th1 lymphocytes are pivotal in the defense against mycobacteria and certain viral microorganisms and are believed to promote ocular inflammatory disorders by triggering effector cytokines such as interleukin (IL)-2 and interferon gamma (IFN-γ). Th2 immune response is linked to protection against helminths and bacteria, whereas the regulatory cytokines IL-4 and IL-5 have prominent roles in allergic diseases [15,16,17].

Some studies have shown an increase in tears cytokines levels in a number of ocular conditions, including allergic conjunctivitis [15,16,17,18,19], dry eye syndrome [18,20,21] and cigarette smoke exposure [22,23]. Previous studies have shown that exposure to air pollution enhances cytokine production in the upper respiratory tract by eliciting both local and systemic inflammatory responses [24,25,26]. However, no study in humans has yet investigated the effect of ambient air pollution on inflammatory tear cytokines levels, *in vivo*. Therefore, the purpose of the present study was to investigate the effect of air pollution levels on tear cytokine levels in subjects from a large metropolitan area exposed to different levels of ambient air pollution.

## Materials and Methods

### Study area and population

This study was conducted according to the Declaration of Helsinki and was approved by the Ethical Committee of Clinical Hospital of University of São Paulo Medical School, São Paulo, Brazil (No.154/2001). All the individuals gave their written informed consent prior to participating in the study.

The study involved 30 subjects aged 31–65 years, evaluated twice, with an interval of 15 days to obtain measurements of the same participant at two different moments in time. They were divided in two groups that worked in different outdoor areas in the São Paulo, the largest metropolis in Brazil and one of the largest cities in Latin America, with high levels of emission from motor vehicles and industries [27]. Group 1 was composed of 19 traffic professionals (taxi drivers or traffic controllers) presumably exposed to high levels of air pollution. Group 2 consisted of 11 workers at the Forest Institute of São Paulo, a protected area of Atlantic rain forest used for recreation and environmental education, and distant about 15 km from downtown São Paulo. Subjects in Group 2 are presumably exposed to somewhat lower levels of air pollution when compared to Group 1. The Forest Institute workers performed occupational activities related to management or environmental research and education. The study excluded workers who performed occupational activities involving the use of chemicals, dusts, mists, fumes, gases or vapors.

Participants of both groups were required to have lived in São Paulo for the last five years to be included in the study. The workers from Group 1 lived in different areas inside the metropolitan area of São Paulo, and those from Group 2 lived nearby the Forest Institute of São Paulo. Other exclusion criteria included: smoking, previous history of ocular surface disease, use of contact lens wear or use of topical ocular drugs.

### Air pollutant exposure assessment

The sampler, designed by the Harvard School of Public Health, operates at a flow rate of 4 lpm through an impaction plate to obtain a PM_2.5_ cutoff. A silicone catheter connects the air inlet positioned at the volunteer shoulder to the sampler inflow. A vacuum pump powered by a rechargeable Li-Ion battery, model WR-5000 Aircheck from SKC, that incorporates a flow control and a chronometer, provides the necessary continuous air flow during the 24-hour sampling period. A polycarbonate membrane, a Whatman filter with a diameter of 37 mm and 0.8 pore size, installed inside the sampler after the impaction plate retains the particulate matter sampled. This membrane was weighed before and after the sampling process in an accurate 1 microgram Mettler Toledo scale, model UMX2, following a laboratorial protocol developed to control temperature and humidity [28], estimating the total mass of particulate matter collected. The volume of air sampled was obtained considering the 4 lpm pump flow and the sampling period recorded by the pump chronometer. The average daily concentration of PM_2.5_ was obtained dividing total mass by total sampled air volume.

The subjects received the samplers at 8 a.m.; were instructed to carry them at all times during their daily activities in a regular day of work and to place them by the bedside at night (monitoring was carried out for 24 h). In the next day, the samplers were returned to the researcher and the filters from the samplers were removed and stored at room temperature, packaged without contact with air, to be analyzed subsequently.

### Tear collection

To measure the inflammatory cytokines levels, tear samples were collected from the inferior lateral meniscus using special glass microcapillaries. (Drummond Scientific Company, Broomall, Pennsylvania, USA). A total of 10 μL was collected from both eyes without anesthesia and transferred to 0.2 mL sterile microtubes. The samples were subsequently stored in a freezer at -80°C. Since time of the day may affect tear cytokine levels [29], all tear samples were collected between 11 and 12 am, at the same outpatient facility. Ambient temperature ranged from 21° to 24°C and air humidity from 40 to 60%.

### Th1 and Th2 cytokines multiplex analysis

IL-2, IL-4, IL-5 and IL-10 and IFN-γ levels were measured using a commercial kit (Th1/Th2 Human 5-Plex Panel, Life Technologies, Grand Island, New York, USA) for analysis in the Bioplex® 200 System (Bio-Rad, Hercules, California, USA). The technique makes it possible to test small aliquots of biological fluids by simultaneously detecting multiple cytokines in a single sample using color-coded beads coated with specific antibodies.

Initially, a mixture of cytokine antibody-specific beads was added to a 96-well filter plate containing standards and tear samples. The tear samples were previously diluted 10-fold with the assay buffer to achieve a final volume of 25 μL. Subsequently, the plate was incubated overnight in an orbital shaker at 500–600 rpm. Streptavidin/R-PE conjugate was added, followed by incubation for 2 hours and detection of bead-specific R-PE fluorescence in a Bio-Plex system.

### Data Analysis

Descriptive statistics included mean ± SD for normally distributed variables or median and interquartile range for non-normally distributed variables. Analysis of histograms and the Kolmogorov-Smirnov test were used to evaluate the normality assumption. The mean values of the two evaluations were considered for cytokines levels and comparisons between the groups were performed by unpaired Student’s t-test or Mann-Whitney test. Individual PM_2.5_ levels were categorized in tertiles (lower, middle and upper) and compared using one-way ANOVA.

To estimate the PM_2.5_ effects on cytokines levels (dependent variables), adjusted for age, years of working and presence of Diabetes Mellitus we have adopted generalized estimating equations (GEE) models considering fixed effects for repeated measures. For this statistical method, we have included both cytokines levels data of evaluations 1 and 2 separately and not the mean values of them. Zeger and Liang [30] have proposed this method for analysis of longitudinal data or repeated measures where serial measurements of a specific variable need to be performed over a period of time. Changes in cytokine levels were presented for increases of 50 μg/m^3^ in PM_2.5_ concentration.

For all statistical tests, type I error for significance was set as *p* <0.05. The statistical analyses were carried out with the SPSS v. 19.0 software (SPSS Inc., Chicago, Il., USA).

## Results

The mean age ± standard deviation of the subjects was 47.8 ± 10.4 years in Group 1 (taxi drivers or traffic controllers) and 50.3 ± 7.1 years in Group 2 (Forest Institute workers) (*p* = 0.38). Diabetes, defined according to the criteria of the American Association of Clinical Endocrinologists Medical Guidelines for Clinical Practice [31] was present in 1 of 22 of traffic professionals and 2 of 11 Forest Institute workers. Fasting blood sugar was determined in all subjects and diabetic subjects were under treatment. In one, fasting plasma glucose level was elevated (156 mg/dL) while all others it was within normal range.

**Fig 1:**
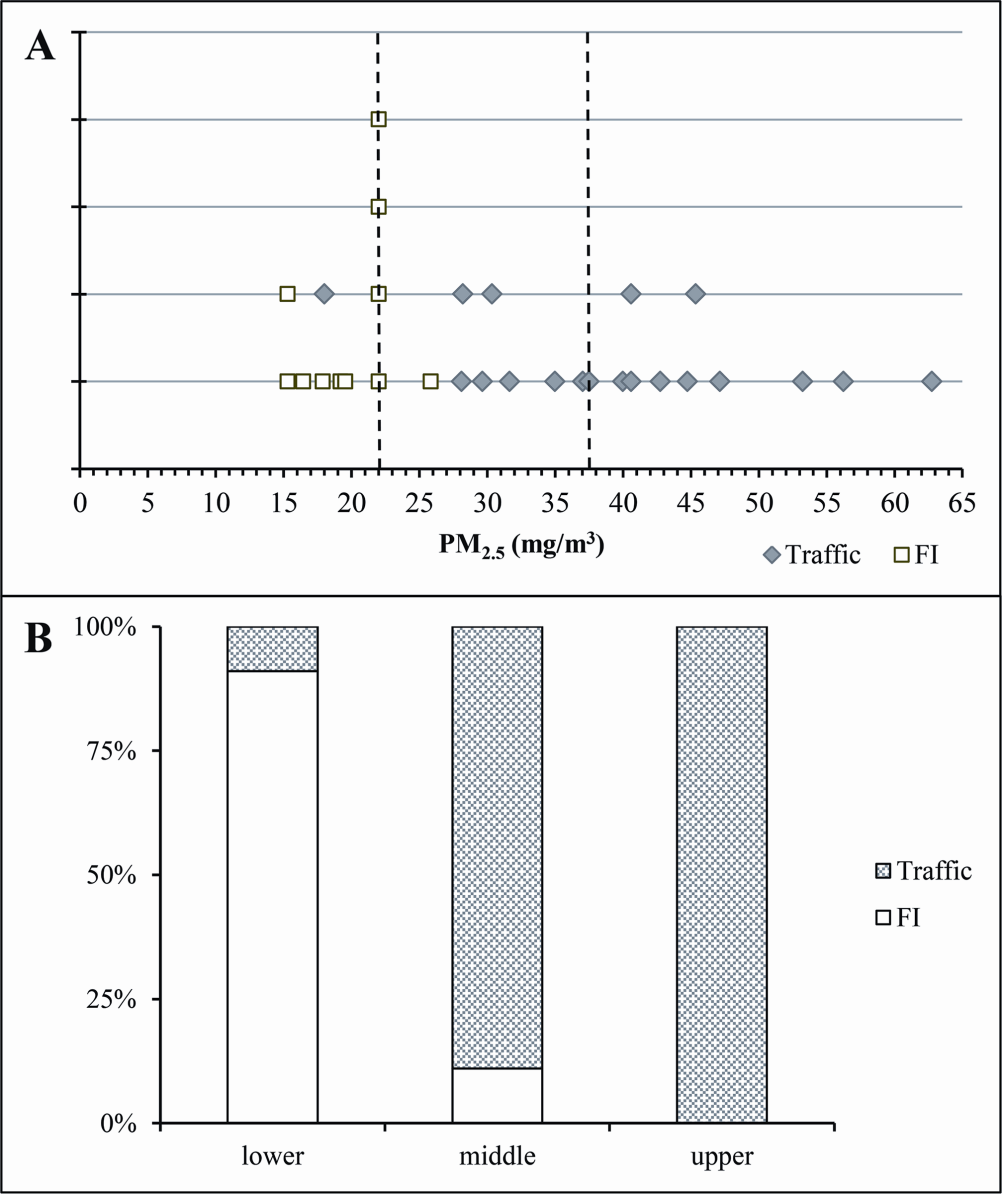
Distribution of subjects in the three categories of PM_2.5_ levels. (A) Dot-plot of individual PM_2.5_ levels (μg/m^3^) of traffic professionals and forest institute staffers (FI). Each data point represents the average of two measurements 15 days apart. PM_2.5_ levels were defined in the three categories (lower: ≤22 μg/m^3^; middle: 23–37.5 μg/m^3^; upper: >37.5 μg/m^3^). (B) Bar graphs showing the percentage distribution of subjects in the three PM_2.5_ categories.* *p*<0.001

Fig 1A is a dot plot of individual PM_2.5_ levels (μg/m^3^) of traffic professionals and forest institute staffers. Each data point represents the average of two measurements 15 days apart. PM_2.5_ levels in the three categories differed significantly (lower: ≤22 μg/m^3^; middle: 23–37.5 μg/m^3^; upper: >37.5 μg/m^3^) (*p*<0.001). Fig 1B shows the percentage distribution of subjects in the three PM_2.5_ categories.

Table 1 shows mean tear cytokine levels in Group 1 and Group 2. IL-2, IL-5, IL-10 and IFN-γ were measurable in tear samples in both groups, but IL-4 level was not detected in any of our samples. No significant differences in tear cytokine levels were found between the groups or between the evaluations.

**Table 1:** Interleukins (IL) 2, 5, 10 and interferon gamma (IFN-γ) tear levels in traffic professionals and Forest Institute workers.

Cytokines (pg/mL)	Traffic professionals	Forest Institute workers	*p*-Value*
**IL-2**	9.09 ± 5.22	10.62 ± 3.93	0.35
**IL-5**	1.07 (1.03)	0.96 (0.77)	0.86
**IL-10**	0.39 (0.37)	0.29 (0.92)	0.67
**IFN-γ**	2.53 ± 1.37	3.10 ± 2.29	0.36

* Unpaired t-test or Mann Whitney test. Data were expressed as mean ± standard deviation or median (interquartile range).

The results of the effects of PM_2.5_ levels on tear cytokine levels are shown in Table 2. In Group 1, a 50μg/m^3^ increment in PM_2.5_ levels was associated with a 1.65 pg/mL decrease in IL-5 levels (*p* = 0.01) and a 0.78 pg/mL decrease in IL-10 levels (*p* = 0.003), but no significant correlation was found between PM_2.5_ levels and IL-2 or IFN-γ levels. In Group 2, no significant correlation was found between PM_2.5_ levels and cytokine levels.

**Table 2:** Effects on interleukins (IL) tear levels 2, 5, 10 and interferon gamma (IFN-γ) in traffic professionals and Forest Institute workers due to a 50μg/m^3^ increment in PM_2.5_ levels.

		Effects on cytokine levels (95% CI)			
Dependent variables	Independent variables	Traffic professionals	*p*-Value*	Forest Institute workers	*p*-Value*
**IL-2 (pg/mL)**	PM_2.5_	-0.72 (-4.95 to 3.51)	0.37	18.70 (-508.87 to 546.28)	0.47
**IL-5 (pg/mL)**	PM_2.5_	-1.65 (-3.12 to -0.17)	*0*.*01*	3.60 (-1.78 to 8.97)	0.09
**IL-10 (pg/mL)**	PM_2.5_	-0.78 (-1.35 to -0.22)	*0*.*003*	0.91 (-3.03 to 4.86)	0.33
**IFN-γ (pg/mL)**	PM_2.5_	-1.31 (-3.46 to 0.83)	0.11	5.69 (-13.96 to 25.34)	0.28

* Generalized estimated equations (GEE) adjusted for age, years of working and presence of Diabetes Mellitus. P values indicated. Significant values are in italics.

## Discussion

The purpose of this study was to evaluate the effect of air pollution on the integrity of the ocular surface system by measuring tear cytokine levels in subjects exposed to different levels of air pollution in a metropolitan setting. It showed that PM_2.5_ levels modulate inflammatory mediators release in the lacrimal film, which can affect the immune defense response of the ocular mucosa.

The PM_2.5_ concentrations for traffic professionals found in our study were above the daily limit of 25 μg/m^3^ specified in WHO air quality guidelines [32]. As expected, traffic professionals (Group 1) were exposed to higher PM_2.5_ levels than Forest Institute staffers (Group 2), presumably due to the nature of their work environment. Although the work environment of the forest institute staffers was less polluted than that of the traffic professionals, they were not living and working in a clean ambient. The geographical location of the forest conservation institute (on the northern outskirts of the city) may still have exposed staffers to relatively middle levels of air pollution. Furthermore, staffers residing in central urban areas may have been exposed to more heavily polluted environments in their free time. Although some of the forest institute staffers had been exposed to PM_2.5_ levels near the maximum daily limit, the group may be considered an acceptable control group for the purpose of the present study. While having a different control population from completely unpolluted areas would be desirable, finding such a group is not an easy task. For example, control groups based on populations in rural areas near São Paulo might be tainted by exposure to emissions from seasonal sugar cane burning [33,34] (a common practice in Brazil), while samples from further removed Brazilian regions would be compromised by geographical and climatic confounding factors, such as differences in temperature, humidity and solar radiation.

In our study, all tear cytokine levels (with the exception of IL-4) could be accurately determined, but no significant variation was observed in any comparison (Table 1). The lack of a significant difference between traffic professionals and forest institute staffers may be explained by the fact that, despite the observed differences in PM_2.5_ levels, both groups were observed into the lower and middle categories of PM_2.5_ levels. For adjustments of interference variables (such as the age, the number of years of working and the presence of Diabetes Mellitus), we have adopted another statistical method, generalized estimating equation (GEE). Using the GEE to estimate the effects of PM_2.5_ levels on cytokine levels, we have found a significant relationship between the two sets of measurements in the group of traffic professionals. A significant reduction on IL-5 and IL-10 tear levels was found for each increments of 50 μg/m^3^ of PM_2.5_ measurement in the air.

Also, it is important to note that the cytokine levels in our study differ from those of other authors. Compared to Carreno et al. [14], who studied normal cytokine levels in tears of healthy subjects using the same methods of lacrimal fluid collection and cytokine analysis adopted in the present study, our subjects had 5 times lower IL-2 levels, 60–100 times lower IL-5 and IL-10 levels, and 15–17 times lower IFN-γ levels. Our cytokine tear levels were also smaller than control values in several previous studies [16,21,35,36]. Differences in tear sampling collection [37], time of the day [29] and in methodology [37] may contribute for explaining the disparity in tear film cytokines values in different studies.

Most studies on tear cytokine levels have focused on patients with dry eye or allergic diseases. In both conditions, tear cytokine levels tend to increase, especially when ocular symptoms are aggravating [15,16,17,18,19,20,21,36,38,39,40]. IL-1, IL-6 and TNF-α are the main Th1-type cytokines associated with dry eye syndrome, [21,38,39] while the inflammatory Th2-type cytokines IL-4, IL-5 and IFN-γ tend to be increased in allergic diseases [15,16,17].

To our knowledge, only one previous study has directly evaluated the effects of air pollution on the ocular surface immune system. Lee et al. [41] investigated the effect of ozone (a byproduct of the photochemical reaction of nitrogen dioxide emitted by motor vehicles) on the tear cytokines in a murine exposure model. The authors found increased levels of IL-1β, IL-6, IL-17 and IFN-γ in mice exposed for 1 or 2 weeks in ozone chambers. In contrast, cytokine levels decreased in our subjects. This discrepancy may be related to differences between environmental and laboratory conditions: although our subjects were exposed to relatively high ambient pollution levels, approximately 1.5 times the recommended upper limit set by WHO [32], the laboratory animals in Lee et al. [41] were submitted to an extremely elevated concentration of pollutants (0.5 and 2 ppm ozone, equivalent to 12 and 50 times the recommended upper limit). As suggested by the authors, exposure to extremely high ozone concentrations seems to interfere with ocular surface integrity by inducing inflammation involving NF-κB-mediated processes, thereby increasing tear cytokine levels. The same study found that conjunctival goblet cells, which protect the ocular surface by synthesizing, storing, and secreting gel-forming mucins in response to external stimuli, were also significantly damaged and lost after ozone exposure, suggesting a significant injury to the ocular surface integrity [41]. Recently, Tau et al. [42] evaluated, *in vitro*, TNF-alpha, IL-6 and IL-8 levels in human corneal (HCLE) and conjunctiva (IOBA) culture cells after acute exposure (24h) to different concentrations of diesel particles (DEP) obtained from the exhaust process of Brazil’s diesel vehicles fleet. The study showed increase in IL-6 levels and a decrease in IL-8 levels. IL-6 plays a key role in the inflammatory acute phase response along with other acute phase proteins such as IL-1β, TNF-α, IFN-γ, TGF-β and IL-8 [43]. IL-6 is also important in the transition from the acute to the chronic inflammatory phase [43]. In the current study, we have studied IL-2, IL-5, IL-10, IFN-γ which are indicative of both acute and chronic inflammatory responses. Our data showed a decrease in IL-5 and IL-10 levels in the group exposed to higher levels of air pollution (PM_2.5_). The apparently discrepant findings between the two studies may be related to the fact that taxi drivers and traffic controllers are chronically exposed to high levels of air pollutants while in Tau´s study evaluated the response to acute air pollutants exposure. Therefore, different immune pathways may have been involved in each of these scenarios.

One possible explanation for the finding of reduced tear cytokine levels is that, as healthy individuals, our subjects were capable of presenting an adaptive ocular response to ambient air pollution. In fact, conjunctival goblet cell hyperplasia (presumably also an adaptive response) and excessive tearing have also been observed in subjects exposed to urban air pollution [7], petrol fumes [44] and smoke from biomass burning [33,34]. Therefore, adaptive tearing could explain the low levels of cytokines observed in our samples, as suggested by Torricelli et al. [5] who found reduced tear osmolarity levels in a similar cohort of normal subjects exposed to high levels of ambient air pollution.

The consequences of low levels of ocular cytokines in healthy individuals are largely unknown. The adaptive response of tearing and reduction of tear film cytokines could be an adaptive strategy of the ocular surface in individuals chronically exposed to air pollution. On the other hand, immune system impairment may also increase susceptibility to systemic diseases in previously healthy subjects. For example, overweight postmenopausal women living in proximity to heavily trafficked roads have been found to have fewer cells in the peripheral blood with the ability to kill cancerous target cells and prevent both cancer and infection than matched controls living far from roadways [45]. Likewise, laboratory experiments have shown an association between exposure to diesel exhaust particles and increased incidence of infection. The repeated inhalation of such particles not only suppressed alveolar macrophages and T cell-mediated immune response to induced Listeria infection in rats [46], but also suppressed the *in vitro* production of IFN-γ, TNF-α, IL-1β and IL-6 by peripheral blood monocytes from healthy individuals [47]. However, further studies are necessary to better understand the tear cytokine response to high air pollution levels exposure.

Our study has some limitations including differences in methodology when compared to other studies in the literature and the fact that some individuals of our control group, although less exposed, were also submitted to PM_2.5_ levels near the maximum daily limit. Despite such limitations, our findings suggest that exposure to high levels of PM_2.5_ reduces tear IL-5 and IL-10 levels that could possibly be an indicator of an ocular immune response modulatory effect. Future studies, however, are necessary to confirm these findings and clarify the mechanisms involved in the immune response of the ocular surface defense system.

## Supporting Information

S1 Dataset Individual PM_2.5_ and cytokine levels of outdoor workers exposed to different levels of ambient air pollution (XLSX)
